# The Therapeutic Effect of Combination of Orbital Decompression Surgery and Methylprednisolone Pulse Therapy on Patients with Bilateral Dysthyroid Optic Neuropathy

**DOI:** 10.1155/2020/9323450

**Published:** 2020-02-19

**Authors:** Jianan Xu, Huijing Ye, Guo Chen, Jingqiao Chen, Rongxin Chen, Huasheng Yang

**Affiliations:** From the State Key Laboratory of Ophthalmology, Zhongshan Ophthalmic Center, Sun Yat-sen University, Guangzhou, China

## Abstract

**Purpose:**

To investigate the synergic effect of combination of orbital decompression surgery and methylprednisolone pulse therapy (MPT) and MPT alone on the visual function in patients with bilateral dysthyroid optic neuropathy (DON).

**Methods:**

For each involved patient with bilateral DON, only one eye was treated with orbital decompression surgery which was conducted by the same doctor, and each of them received MPT after surgery. If the visual function deteriorated despite treatment, patients would switch to the other treatment. All the patients were followed up for 3 months after surgery. Clinical features of patients including best corrected visual acuity (BCVA), intraocular pressure (IOP), proptosis, upper eyelid retraction, and clinical activity score (CAS) before and after surgery were analyzed, respectively. Visual field and visual evoked potential (VEP) tests were also performed. Paired *t*-test and Wilcoxon matched-pairs signed ranks sum test were used to analyze the data.

**Result:**

A prospective cohort of 23 patients with bilateral DON was enrolled in this cohort study. No patients failed to the therapy or switched to another treatment. The quantitative variables were shown as means and standard deviations (SD). After 3 months of combined treatment of orbital decompression surgery and MPT, BCVA (logMAR) improved, proptosis was reduced and the upper eyelid retraction was relieved in both eyes of patients; however, these improvements were more significant in the operated eyes than in the fellow (nonoperated) eyes. IOP decreased significantly in the operated eyes (*P*=0.002), while having no significant change in the nonoperated eyes (*P*=0.002), while having no significant change in the nonoperated eyes (*P*=0.002), while having no significant change in the nonoperated eyes (*P*=0.002), while having no significant change in the nonoperated eyes (*P*=0.002), while having no significant change in the nonoperated eyes (*P*=0.002), while having no significant change in the nonoperated eyes (*P*=0.002), while having no significant change in the nonoperated eyes (*P*=0.002), while having no significant change in the nonoperated eyes (*P*=0.002), while having no significant change in the nonoperated eyes (*P*=0.002), while having no significant change in the nonoperated eyes (*P*=0.002), while having no significant change in the nonoperated eyes (*P*=0.002), while having no significant change in the nonoperated eyes (*P*=0.002), while having no significant change in the nonoperated eyes (*P*=0.002), while having no significant change in the nonoperated eyes (

**Conclusion:**

A combination of orbital decompression and MPT can significantly improve visual function in patients with DON, reduce intraocular pressure, and relieve clinical symptoms such as upper eyelid retraction and proptosis, while MPT alone has a limited effect. For DON patients, orbital decompression should be performed promptly to improve the visual function.

## 1. Introduction

Thyroid associated ophthalmopathy (TAO) is an orbital lesion accompanied with a functional disorder of thyroid endocrine axis [1] and has the highest incidence among adults with orbital disorders [2]. Dysthyroid optic neuropathy (DON) is a serious complication of TAO and accounts for 4–8% of TAO patients [3]. It occurs more often among men than women and more frequently among older patients, especially those with diabetes [4]. The pathogenesis of DON includes multiple factors. Bulging of orbital connective tissue and fat leads to hypertrophy of extraocular muscle and obstruction of venous return which in turn induces high intraorbital pressure and decreased vessel density in the peripapillary area [5]. Orbital crowding may also result in increasing pressure in the episcleral veins, which may lead to increasing intraocular pressure (IOP) and optic nerve compression [6]. As a shield, the orbital septum limits the expansion of the intraorbital tissue and aggravates the increase of intraorbital pressure. Once the narrow orbital apex is affected, the optic neuropathy may become more serious. In addition, the hypertrophic extraocular muscle wrapping around the optic nerve will cause annular compression [7]. These factors can interact with each other, resulting in compression of the optic nerve and vessels responsible for ocular reperfusion, such as ophthalmic vein, central retinal artery, and posterior ciliary artery, which in turn leads to ischemia and irreversible visual impairment [8].

DON is difficult to diagnose at the early stage, and half of the patients with optic neuropathy fail to realize its existence; delay in its treatment tremendously impairs the patients' visual function and quality of their life [9, 10]. So far, there have been numerous studies on the clinical characteristics, diagnosis, and treatment of DON. Recent research studies have shown that the current evidence is sufficient to support intravenous glucocorticoids (IVGC) to be the first-line treatment for moderate-to-severe TAO, and the use of rituximab or mycophenolate mofetil (MMF) and tocilizumab to be the second-line treatment instead of IVGC. However, the evidence is insufficient to support the use of IVGC or orbital decompression as the first-line treatment of DON [11, 12]. Besides, to our best knowledge, there is no relevant study to investigate the respective roles played by surgical operation and hormone therapy in the improvement of visual function of patients with DON treated with a combination of orbital decompression surgery and methylprednisolone pulse therapy (MPT). In this study, only one eye of each patient with bilateral DON was treated with orbital decompression surgery combined with MPT, and the fellow eye received separate MPT without combination with orbital decompression surgery. We compared related clinical characteristics of patients before and after treatment to explore the effect of the combination of orbital decompression surgery and MPT and MPT alone on the DON.

## 2. Materials and Methods

### 2.1. Patients

TAO patients diagnosed with bilateral DON in Zhongshan Ophthalmic Center, Sun Yat-sen University (Guangzhou, China) from January 2016 to January 2019 were enrolled in the study. This study was conducted in accordance with the Declaration of Helsinki and approved by the clinical research ethics committee (protocol #2015MEKY086). All written informed consents were obtained from involved patients.

All patients underwent comprehensive ophthalmic evaluations before treatment and after 3 months treatment, including review of medical history, dioptroscopy, best corrected visual acuity (BCVA) assessment, slit-lamp and fundoscopic examination, IOP measurement by Goldman applanation tonometry, and examination of proptosis, upper eyelid retraction, eye movement, and eye position. The visual field was examined by HFA (Humphrey II, Carl-Zeiss, Dublin, CA). Reliability criteria included fixation losses being less than 20%, false-positive rate being 15% or lower, and false-negative rate being 33% or lower. Visual evoked potentials (VEP) test was examined. The visual stimulus was a pattern reversal checkerboard displayed on a black and white monitor placed 105 cm from the patient. In addition, computed tomography (CT) or magnetic resonance imaging (MRI) was performed.

Severity and activity of all subjects were assessed according to NOSPECS (no physical signs or symptoms, only signs, soft tissue involvement, proptosis, extraocular muscle signs, corneal involvement, and sight loss) grading and clinical activity score (CAS) scoring [13]. Patients with CAS of 3 or higher are defined as active stage, while those with CAS score below 3 are defined as nonactive stage.

### 2.2. Diagnostic and Inclusion Criteria

The diagnostic criteria for DON included (1) BCVA of 20/40 or lower; (2) anomalopia; (3) abnormal visual field results of obviously decreased mean deviation; (4) abnormal VEP test results of severely prolonged latency and reduced amplitude; and (5) CT or MRI showed generalized enlargement of the extraocular muscles and expansion of the orbital fat. All of the criteria listed above were necessary for diagnosis. Patients who met the criteria simultaneously in both eyes were diagnosed with bilateral DON.

The inclusion criteria for patients included (1) diagnosis of sight-threatening TAO with bilateral DON, without other severe eye diseases; (2) no previous intraocular surgeries and methylprednisolone therapy performed at least 6 months ago; (3) DON being the only disease causing visual function defect; and (4) no medical or family history of diabetes mellitus, cerebral diseases, or other optic neuropathy.

### 2.3. Surgical Technique

Bony orbital decompression was performed by the same doctor with rich clinical experience. The surgery was performed under general anesthesia. The transcutaneous approach was used for medial and orbital wall decompression. The arcuate incision was made in the skin 2 mm below the lower eyelid margin with sterile scalpel blade, and the tissue under the incision was separated to the periorbita and orbital septum. Part of the medial orbital wall, inferior orbital wall, and partial tissue of ethmoidal sinus was removed, and an appropriate amount of adipose tissue was excised. MPT was conducted after the surgery, at a dose of 1 g methylprednisolone per day through intravenous injection for 3 consecutive days with a total amount of 3 g methylprednisolone. After 3 days of intravenous injection of methylprednisolone, prednisone was orally taken by the patients at a dose of 30 mg/day, which was gradually reduced.

### 2.4. Statistical Analysis

Statistical analysis was performed using SPSS software (version 21, SPSS Inc., Chicago, IL). The means and standard deviations (SD) of the quantitative variables were calculated. Paired design was conducted. The paired *t*-test was used to detect the differences of quantitative variables when data obeyed normal distribution; otherwise, the Wilcoxon matched-pairs signed ranks sum test was used. *P* < 0.05 was considered to be statistically significant.

## 3. Results

### 3.1. Demographic Data

**Figure 1:**
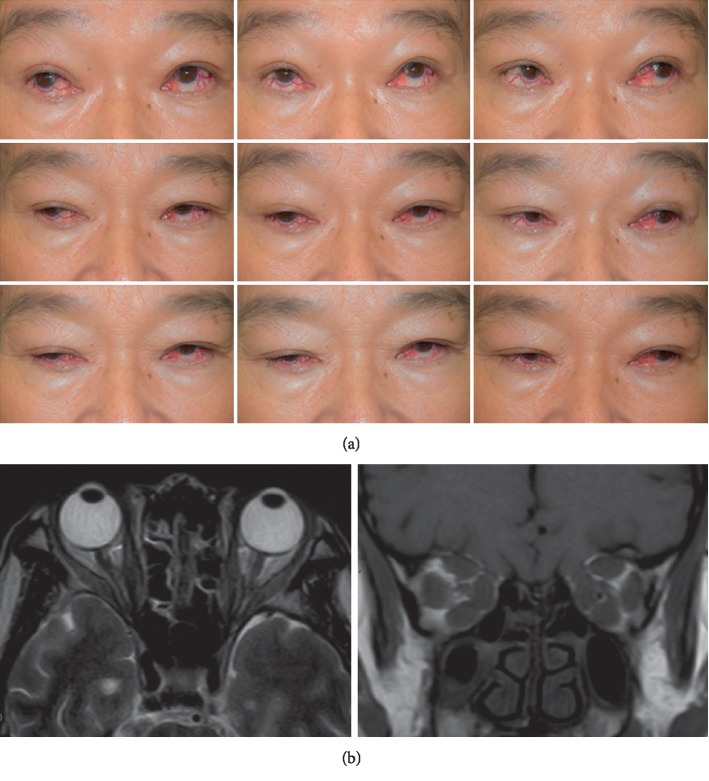
One case diagnosed with bilateral dysthyroid optic neuropathy (DON). (a) Eye movement testing showed limited eye movement and strabismus in both eyes. (b) Magnetic resonance imaging (MRI) showed enlargement of extraocular muscles.

A prospective cohort of 23 patients with bilateral DON who fulfilled the eligibility criteria were enrolled, including 15 males and 8 females, with an average age of 58.3 ± 8.93 years. A case diagnosed with bilateral DON before treatment was shown in Figure 1. The demographic characteristics are shown in Table 1.

### 3.2. Clinical Characteristics

Compared with the corresponding parameters before combined treatment of orbital decompression surgery and MPT, after 3 months treatment BCVA (logMAR) of the operated eyes improved from 1.3 ± 0.63 to 0.7 ± 0.54 (*P* = 0.001), IOP decreased from 20.5 ± 6.21 mmHg to 16.1 ± 4.43 mmHg (*P* = 0.002), proptosis decreased from 20.0 ± 4.24 mm to 15.7 ± 3.00 mm (*P* < 0.001), upper eyelid retraction decreased from 3.1 ± 2.05 mm to 1.9 ± 1.46 mm (*P* < 0.001), and CAS decreased from 1.6 ± 1.37 to 0.8 ± 0.87 (*P* = 0.011). After 3 months of treatment with MPT alone, BCVA (logMAR) of fellow eyes improved from 0.7 ± 0.51 to 0.5 ± 0.43 (*P* = 0.018), IOP was not significantly changed (*P* = 0.993), the proptosis decreased from 19.2 ± 4.18 mm to 17.8 ± 3.84 mm (*P* = 0.008), the upper eyelid retraction decreased from 3.0 ± 2.12 mm to 2.4 ± 1.76 mm (*P* < 0.001), and CAS decreased from 1.5 ± 1.37 to 0.6 ± 0.73 (*P* = 0.005). The comparison of clinical characteristics of the operated eyes and fellow eyes is shown in Table 2.

After 3 months of the combined treatment of orbital decompression surgery and MPT, the improvement of BCVA (logMAR), the decrease of IOP, the reduction of proptosis, and the relief of upper eyelid retraction of the operated eyes were more significant than those of the fellow eyes (*P* < 0.05, respectively). The CAS reduced by 0.8 ± 1.37 in the operated eyes and by 0.9 ± 1.28 in the fellow eyes, but the reduction extent of CAS showed no significant difference between the operated and fellow eyes (*P*=0.771). The comparison of the improvement of clinical parameters between the operated eyes and fellow eyes is shown in Table 3.

### 3.3. Visual Field Parameters

After 3 months of combined treatment of orbital decompression surgery and MPT, the mean deviation (MD) of the operated eyes increased significantly from −19.8 ± 9.90 dB to −11.7 ± 8.82 dB (*P* < 0.001), but the pattern standard deviation (PSD) of the operated eyes showed no significant difference (*P*=0.206), compared with that before treatment. After 3 months of treatment with MPT alone, the MD of fellow eyes increased significantly from −12.8 ± 7.45 dB to −9.4 ± 6.98 dB (*P*=0.001), and the PSD of fellow eyes showed no significant change (*P*=0.246). The comparison of visual field parameters in the operated eyes and fellow eyes is shown in Table 4.

**Figure 2:**
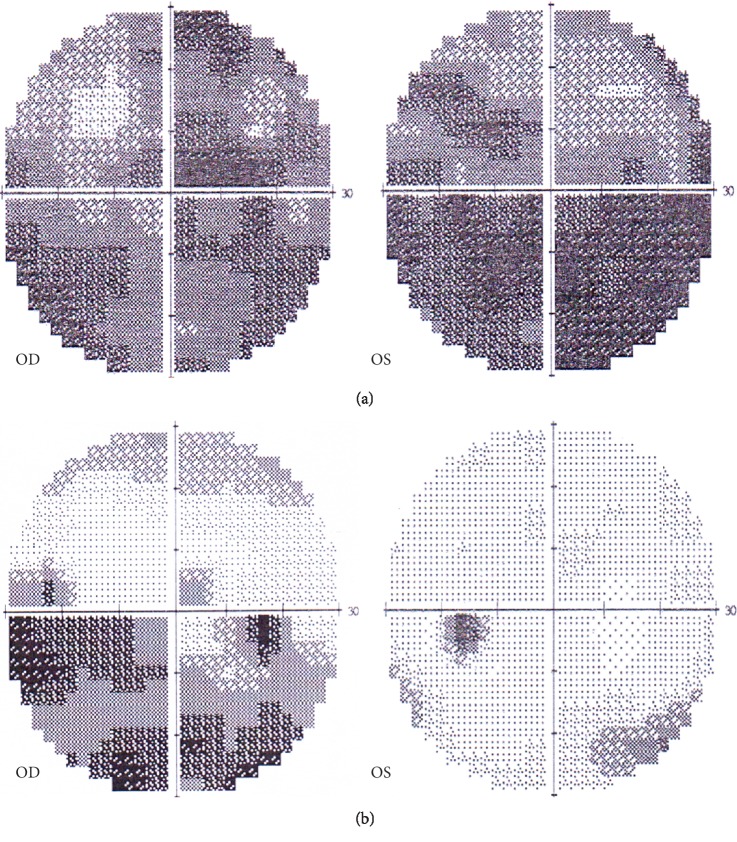
Grayscale showed visual field defect before and after orbital decompression on the left eye combined with methylprednisolone pulse therapy (MPT). (a) Severe visual field defect was shown in both eyes before orbital decompression on the left eye combined with MPT. (b) Visual field defect relieved in both eyes after orbital decompression on the left eye combined with MPT (the improvement in the operated eye was more significant than that of the fellow eye).

After 3 months of combined treatment of orbital decompression surgery and MPT, the MD in the operated eyes improved by 8.1 ± 7.72 dB, more significant than that of the fellow eyes (*P*=0.005). No significant difference was found in the PSD improvement between the operated eyes and the fellow eyes (*P*=0.852). The comparison of improvement of visual field parameters of the operated eyes and fellow eyes is shown in Table 5. Visual field defects of one patient before and after orbital decompression on the left eye combined with MPT are shown in Figure 2.

### 3.4. VEP Parameters

Compared with the corresponding parameters before combined treatment of orbital decompression surgery and MPT, the latency of 60′ P100 in operated eyes decreased from 166.3 ± 38.98 ms to 138.2 ± 37.08 ms (*P*=0.002), that of 30′ P100 decreased from 186.2 ± 26.84 ms to 145.9 ± 33.82 ms (*P*=0.001), and that of 15′ P100 decreased from 187.5 ± 25.19 ms to 166.8 ± 31.55 ms (*P*=0.005); the amplitude of 60′ P100 increased from 3.8 ± 3.58 *μ*V to 6.3 ± 4.05 *μ*V (*P*=0.002), that of 30′ P100 increased from 2.1 ± 3.16 *μ*V to 4.5 ± 3.51 *μ*V (*P*=0.001), and that of 15′ P100 increased from 2.1 ± 3.53 *μ*V to 4.2 ± 4.97 *μ*V (*P*=0.021). After the separate MPT without combination of orbital decompression surgery, VEP parameters of nonoperated eyes showed no significant change (*P* > 0.05, respectively). The comparison of VEP parameters of the operated eyes and fellow eyes is shown in Table 6.

**Figure 3:**
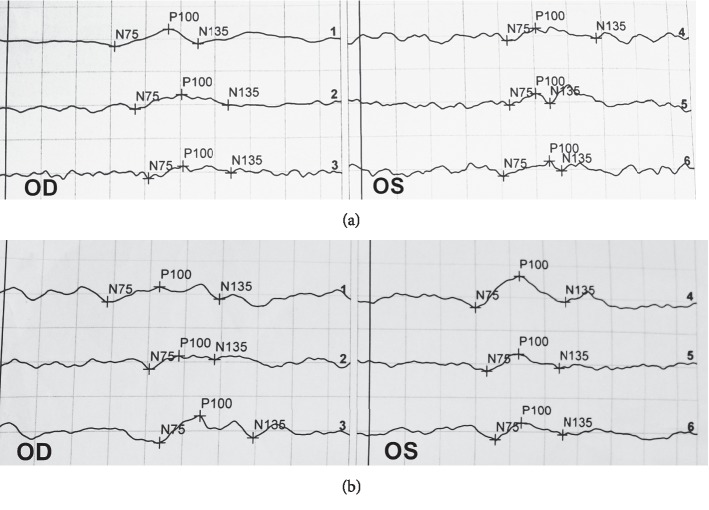
Visual evoked potentials (VEP) testing showed latencies and amplitudes before and after orbital decompression on the left eye combined with methylprednisolone pulse therapy (MPT). (a) Prolonged latencies and reduced amplitudes were shown in both eyes before orbital decompression on the left eye combined with MPT. (b) Latencies and amplitudes restored in operated eyes after orbital decompression on the left eye combined with MPT.

After 3 months of combined treatment of orbital decompression surgery and MPT, the latency of 60′ P100 in the operated eyes improved by 28.1 ± 29.93 ms, the latency of 30′ P100 improved by 40.2 ± 32.87 ms, and the latency of 15′ P100 improved by 20.7 ± 25.87 ms, which was more excellent in the degree of the improvement than that in the fellow eyes (*P*=0.002, *P*=0.001, and *P*=0.005, respectively). The comparison of improvement of VEP parameters of the operated eyes and fellow eyes is shown in Table 7. Latencies and amplitudes of VEP testing of one patient before and after orbital decompression on the left eye combined with MPT are shown in Figure 3.

### 3.5. Side Effects

All the 23 DON patients received complete treatment and no patients failed to the therapy or switched to another treatment. No serious complications such as cerebrospinal fluid leak, lacrimal system injury, or vision loss were encountered. Side effects consisted of weight gain in 5 patients, Cushing syndrome appearance in 2 patients, transient diplopia of the in 3 patients, and new onset strabismus in 1 patient after 3 months of treatment.

## 4. Discussion

As an organ-specific disease closely related to autoimmunity, TAO has very complex clinical manifestations, deteriorating quickly and affecting the quality of life of patients [14]. For patients of TAO with different severity and at different stages, as well as those with severe complications like DON, the benefits and side effects of treatment need to be comprehensively considered and balanced to achieve optimum effects. Previous studies have suggested that MPT appears to be the first choice for active TAO with DON rather than immediate surgery, while a complete recovery can be achieved in the majority of patients with DON by combination therapy [15–18]. Numerous researches have investigated the clinical manifestations, diagnosis, and treatment of TAO so far, but researches on the benefits and timing of decompression surgery and MPT for DON patients are deficient [17, 19–21]. This study investigated and compared the effect of combined treatment of orbital decompression and MPT on one eye of patients with bilateral DON, and the effect of separate MPT on the fellow eye of the same patients.

For TAO patients complicated with DON, MPT is proposed as a first-line treatment by EUGOGO [13]. However, for those insensitive to MPT and at the advanced stage of the disease, decompression surgery is recommended to prevent deterioration of visual function. Bernardino et al. found that logMAR visual acuity scores were significantly improved after orbital decompression surgery [22, 23]. In this study, although the visual acuity of fellow eyes treated with MPT alone improved after treatment, the improvement was less significant than in the operated eye.

The IOP of patients with DON can be affected by multiple factors, such as intraorbital pressure and severe strabismus. The current orbital manometry was usually performed using the finger-press method or self-designed orbital manometer [24], which is incapable of distinguishing false-positive results caused by intraorbital pressure and whose reliability and repeatability still need to be confirmed. Besides, strabismus may also lead to false-positive increase in results of IOP measurement caused by abnormal eye position [25]. Therefore, measurement errors arising from increased orbital pressure or strabismus need to be distinguished and excluded. In this study, IOP in patients treated with orbital decompression significantly reduced after surgery, and the reduction extent was consistent with that shown in previous studies [26]. The reason for different effects in IOP between the operated and fellow eyes may be that orbital decompression expands the orbital cavity and reduces the intraorbital pressure.

Orbital decompression can relieve the proptosis and the upper eyelid retraction. Soares-Welch found that the proptosis and the upper eyelid retraction in the eyes treated with orbital decompression were more significantly relieved than those in the fellow eyes treated with MPT only [27]. The Alhambra Expósito team also found that there was also partial relief of proptosis and upper eyelid retraction in the fellow eyes treated with MPT, but the effects were not as significant as in eyes treated with orbital decompression [28]. The possible reason may be that the removal of part of the orbital wall enlarges the orbital cavity and removes part of the adipose tissue, alleviating the pressure on the anterior orbital of muscles and soft tissues [29].

According to previous studies, in terms of activity indicators, the average CAS of patients, which was 3.8 before surgery, decreased significantly after orbital decompression surgery [30]. In this study, there was no significant difference in the reduction extent between the operated and fellow eyes, mainly due to the systemic effect of MPT on both eyes. However, as most of the involved patients were at the nonactive stage of TAO before surgery, the reduction extent of postoperative CAS in this study was not as significant as that in other studies.

Visual functions such as VEP and visual field tests are also important for the diagnosis of DON [31]. As an objective visual electrophysiology analysis, VEP helps to detect early, evasive optic neuropathy. The reduced amplitude of the VEP waveform indicates optic nerve axonal degeneration and prolonged latency often reflects abnormal optic nerve conduction. The pattern of the VEP change in DON patients is still controversial. In their study, Mario Salvi et al. found that in TAO patients, P100 latency was prolonged, but the amplitude did not change significantly [32]. By contrast, Ning Hua compared VEP change in TAO patients with that in normal subjects and found no significant delay in P100 latency in those patients, but the amplitude was significantly reduced, especially in high spatial frequencies [33]. The reason for the delay of P100 latency in DON patients was that the demyelination of optic nerve from pressure and remyelination cause myelin sheath to get thinner, and the nerve impulse conduction changes from saltatory to continuous conduction. The decrease in amplitude is mainly due to the chronic compression injury in neurons and axons, resulting in decreased electrical activity of the optic nerve. Therefore, both P100 latency and amplitude reflect damage in the optic nerve in DON patients [34]. Orbital decompression can alleviate optic nerve demyelination and increase neuronal excitability by reducing optic nerve compression, thereby shortening the P100 latency and partially increasing the P100 amplitude. Low spatial frequency VEP mainly indicates the lesion of the area near the fovea, while the high spatial frequency VEP mainly reflects the lesion of the fovea. Therefore, the results of this study suggest that orbital decompression may be more effective in improving the lesion of the fovea.

Visual field defects of DON patients usually have various symptoms but are generally manifested as central scotoma, which can extend to the peripheral area as the disease develops, have different shapes, and be partially ameliorated after effective treatment. Previous studies have shown that decompression surgery can significantly increase the MD value with an average increase of approximately 9.4 to 10.0 dB [35, 36]. This study revealed that the MD in operated eyes of DON patients improved more significantly than that in the fellow eyes received MPT alone, as also observed in the previous studies. The PSD that indicates the localized visual field loss had no significant difference before and after orbital decompression. We theorized that the diffuse visual field defects in patients before surgery resulted in a low preoperative PSD value; although orbital decompression improved visual field defects generally, it affected optic nerve nonuniformly: in some patients localized visual field defects might get more salient after surgery, leading to higher PSD values while in others the visual field defects were relatively slight after surgery, resulting in lower PSD values. Therefore, the average PSD showed no significant difference after surgery.

In conclusion, this study analyzed and compared the effect of a combination of orbital decompression surgery and MPT, and MPT alone on patients with bilateral DON. A combination of orbital decompression surgery and MPT can significantly improve visual function in patients with DON, reduce intraocular pressure, and relieve symptoms such as upper eyelid retraction and proptosis, while MPT alone has a limited effect. For DON, orbital decompression surgery should be performed promptly to improve the visual function.

## Figures and Tables

**Table 1:** Demographic characteristics of the patients with bilateral DON.

	Bilateral DON *N* = 23
Age (year)	58.3 ± 8.93
Gender (M/F)	15/8
Thyroid function	
Hyperthyroidism (%)	30.4
Euthyroidism (%)	43.5
Hypothyroidism (%)	26.1
Duration of TAO (month)	28.5 ± 23.70

DON: dysthyroid optic neuropathy; M: male; F: female; TAO: thyroid associated ophthalmopathy.

**Table 2:** Clinical characteristics of the operated eyes and fellow eyes in patients with bilateral DON before and after treatment.

	Operated eye, *N* = 23		*P*	Fellow eye, *N* = 23		*P*
	Before treatment	After treatment		Before treatment	After treatment	
BCVA	1.3 ± 0.63	0.7 ± 0.54	0.001^†^	0.7 ± 0.51	0.5 ± 0.43	0.018^†^
IOP (mmHg)	20.5 ± 6.21	16.1 ± 4.43	0.002^†^	18.5 ± 4.80	18.5 ± 7.24	0.993^‡^
Proptosis (mm)	20.0 ± 4.24	15.7 ± 3.00	＜0.001^‡^	19.2 ± 4.18	17.8 ± 3.84	0.008^‡^
Upper eyelid retraction (mm)	3.1 ± 2.05	1.9 ± 1.46	＜0.001^†^	3.0 ± 2.12	2.4 ± 1.76	＜0.001^†^
CAS	1.6 ± 1.37	0.8 ± 0.87	0.011^†^	1.5 ± 1.37	0.6 ± 0.73	0.005^†^

^†^Wilcoxon matched-pairs signed ranks sum test between the eyes before and after treatment. ^‡^Paired *t*-test between the eyes before and after treatment. DON: dysthyroid optic neuropathy; BCVA: best-corrected visual acuity; IOP: intraocular pressure; CAS: clinical activity score.

**Table 3:** Improvement of clinical parameters of the operated eyes and fellow eyes after treatment in patients with bilateral DON.

	Improvement of operated eye, *N* = 23	Improvement of fellow eye, *N* = 23	*P*
BCVA	0.5 ± 0.59	0.2 ± 0.34	0.046^†^
IOP (mmHg)	4.4 ± 6.79	0.0 ± 5.71	0.021^†^
Proptosis (mm)	4.3 ± 2.52	1.4 ± 1.94	＜0.001^‡^
Upper eyelid retraction (mm)	1.3 ± 1.12	0.6 ± 0.67	0.003^†^
CAS	0.8 ± 1.37	0.9 ± 1.28	0.771^†^

^†^Wilcoxon matched-pairs signed ranks sum test between the eyes before and after treatment. ^‡^Paired *t*-test between the eyes before and after treatment. DON: dysthyroid optic neuropathy; BCVA: best corrected visual acuity; IOP: intraocular pressure; CAS: clinical activity score.

**Table 4:** Visual field parameters of the operated eyes and fellow eyes in patients with bilateral DON before and after treatment.

	Operated eye *N* = 23		*P*	Fellow eye *N* = 23		*P*
	Before treatment	After treatment		Before treatment	After treatment	
MD (dB)	−19.8 ± 9.90	−11.7 ± 8.82	＜0.001^†^	−12.8 ± 7.45	−9.4 ± 6.98	0.001^†^
PSD (dB)	5.3 ± 2.81	4.5 ± 2.03	0.206^†^	6.4 ± 2.45	5.7 ± 3.44	0.246^†^

^†^: Wilcoxon matched-pairs signed ranks sum test between the eyes before and after treatment. DON: dysthyroid optic neuropathy; MD: mean deviation; PSD: pattern standard deviation.

**Table 5:** Improvement of visual field parameters of the operated eyes and fellow eyes after treatment in patients with bilateral DON.

	Improvement of operated eye, *N* = 23	Improvement of fellow eye, *N* = 23	*P*
MD (dB)	8.1 ± 7.72	3.4 ± 5.02	0.005^†^
PSD (dB)	0.9 ± 3.53	0.7 ± 3.06	0.852^†^

^†^Wilcoxon matched-pairs signed ranks sum test between the eyes before and after treatment. DON: dysthyroid optic neuropathy; MD: mean deviation; PSD: pattern standard deviation.

**Table 6:** Visual evoked potential parameters of the operated eyes and fellow eyes in patients with bilateral DON before and after treatment.

	Operated eye, *N* = 23		*P*	Fellow eye, *N* = 23		*P*
	Before treatment	After treatment		Before treatment	After treatment	
60′ P100 latency (ms)	166.3 ± 38.98	138.2 ± 37.08	0.002^†^	137.8 ± 39.00	141.1 ± 36.36	0.139^†^
60′ P100 amplitude (*μ*V)	3.8 ± 3.58	6.3 ± 4.05	0.002^†^	7.7 ± 4.39	7.3 ± 6.19	0.278^†^
30′ P100 latency (ms)	186.2 ± 26.84	145.9 ± 33.82	0.001^†^	146.7 ± 32.66	163.9 ± 41.98	0.139^†^
30′ P100 amplitude (*μ*V)	2.1 ± 3.16	4.5 ± 3.51	0.001^†^	5.1 ± 3.22	5.3 ± 4.84	0.926^†^
15′ P100 latency (ms)	187.5 ± 25.19	166.8 ± 31.55	0.005^†^	160.2 ± 33.48	170.1 ± 36.10	0.307^†^
15′ P100 amplitude (*μ*V)	2.1 ± 3.53	4.2 ± 4.97	0.021^†^	5.4 ± 4.40	4.3 ± 4.18	0.172^†^

^†^Wilcoxon matched-pairs signed ranks sum test between the eyes before and after treatment. DON: dysthyroid optic neuropathy.

**Table 7:** Improvement of visual evoked potential parameters of the operated eyes and fellow eyes after treatment in patients with bilateral DON.

	Improvement of operated eye, *N* = 23	Improvement of fellow eye, *N* = 23	*P*
60′ P100 latency (ms)	28.1 ± 29.93	−3.3 ± 10.17	0.002^†^
60′ P100 amplitude (*μ*V)	2.5 ± 3.97	−0.1 ± 5.51	0.102^†^
30′ P100 latency (ms)	40.2 ± 32.87	−17.2 ± 35.55	0.001^†^
30′ P100 amplitude (*μ*V)	2.4 ± 3.56	0.0 ± 4.31	0.083^†^
15′ P100 latency (ms)	20.7 ± 25.87	−9.9 ± 36.30	0.005^†^
15′ P100 amplitude (*μ*V)	2.0 ± 6.18	−1.1 ± 4.49	0.004^†^

^†^Wilcoxon matched-pairs signed ranks sum test in the operated and fellow eyes before and after treatment. DON: dysthyroid optic neuropathy.

## Data Availability

The dataset analyzed during the current study may be available from the corresponding author upon reasonable request.
